# New *PAX2* heterozygous mutation in a child with chronic kidney disease: a case report and review of the literature

**DOI:** 10.1186/s12882-018-1044-9

**Published:** 2018-09-21

**Authors:** Li Zhang, Shu-bo Zhai, Leng-yue Zhao, Yan Zhang, Bai-chao Sun, Qing-shan Ma

**Affiliations:** Department of Pediatrics Nephrology, First Hospital, Jilin University, Changchun, Jilin, 130021 China

**Keywords:** *PAX2* mutation, Chronic kidney disease, Child, Hypoplasia

## Abstract

**Background:**

We herein report a 3-year-old boy presented with chronic kidney disease (CKD) due to *PAX2* missense mutation (C to G transversion at position 418 in exon 4).

**Case presentation:**

He attended our clinic with a 3-month history of foamy urine. Upon examination, he had reduced estimated glomerular filtration rate (GFR) and renal atrophy. Genetic investigations revealed that he has inherited a mutated *PAX2* gene from his father, who had renal failure at the age of 20. We searched the literature and confirmed that this mutation site has not been reported by any other group before.

**Conclusions:**

Although renal coloboma syndrome (RCS) with simultaneous kidney and eye involvement is the most common phenotype of *PAX2* mutations, current literature supports that such mutations may have profuse clinical manifestations and renal hypoplasia is one distinct entity in the spectrum.

## Background

CKD is a serious condition in children that leads to significant morbidity and mortality. The prevalence of end stage renal disease (ESRD) among children aged 19 years and younger in United States was 12.9 per million/year [1]. In contrast to the adult population, obstructive uropathy and congenital renal aplasia/hypoplasia/dysplasia are responsible for almost one half of all cases of CKD in children [2]. Recent studies have shown that *PAX2* gene plays critical roles in organogenesis during embryonic development, and the *PAX2* mutation is the most common cause of renal hypoplasia [3]. The complete ablation of *PAX2* stunts renal organogenesis at early stage of kidney development and can result in kidney agenesis [4]. The most common renal phenotype caused by mutation of *PAX2* is renal coloboma syndrome (OMIM #120330; also known as papillorenal syndrome). In this report, we present a child with CKD caused by renal hypoplasia. Genetic investigation confirmed that he had a mutation in the *PAX2* gene that had not been reported in the literature before.

## Case presentation

**Fig. 1:**
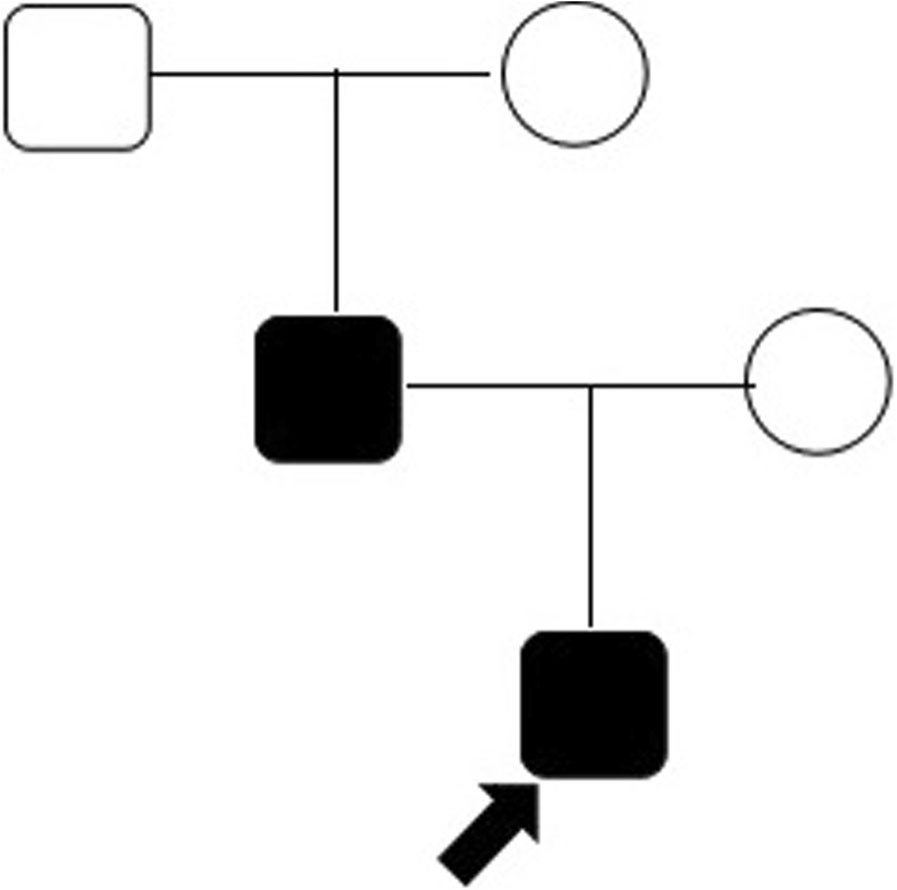
The family pedigree

**Fig. 2:**
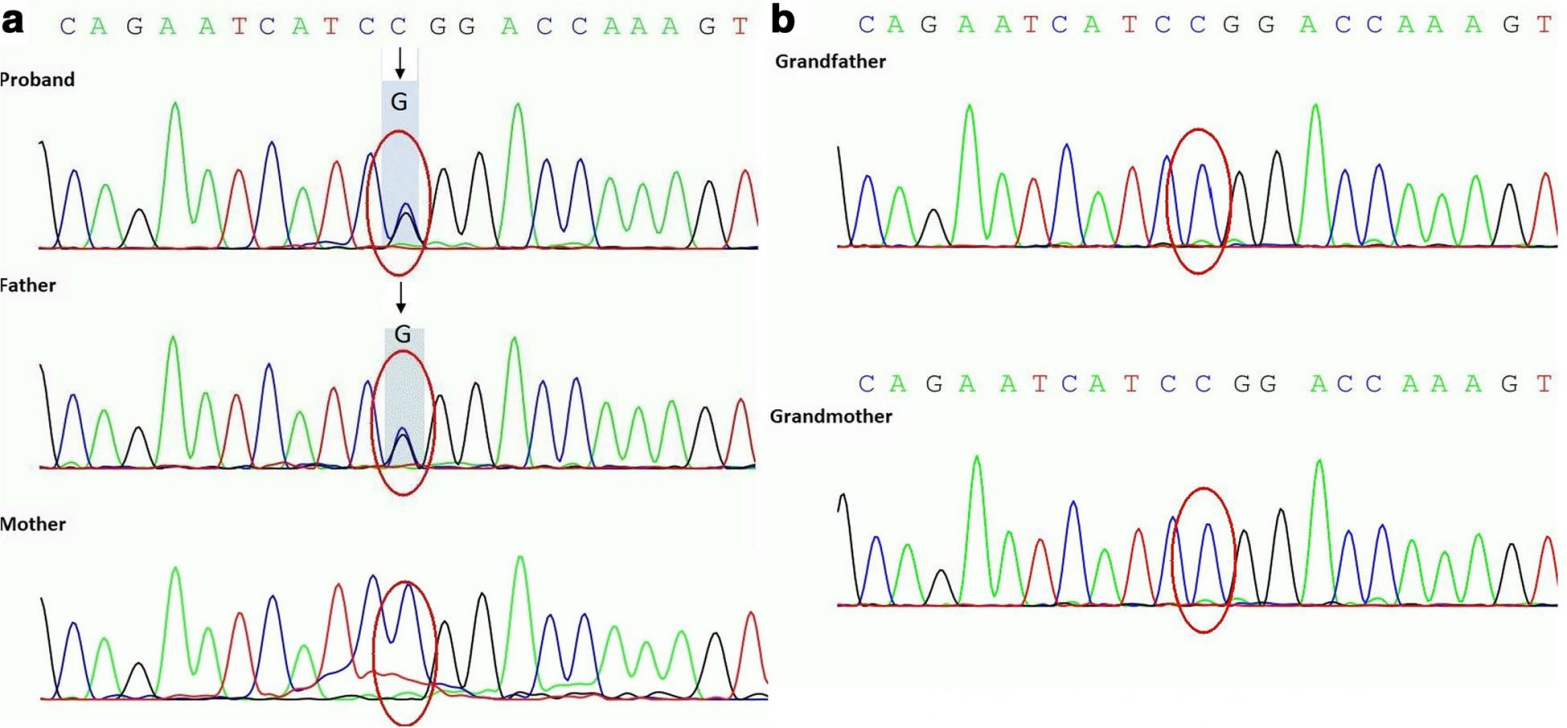
**a** The same *PAX2* mutation in proband and his father. (c.418C>G, p.R140G, exon4, chr10–102,539,262, NM_000278). **b** Normal genotype in paternal grandparents

A 3-year-old boy presented to our hospital with a 3-month history of foamy urine. He was born at gestational age of 36 weeks 6 days to a young couple with no history of consanguinity. Prior history was significant for decreased amniotic fluid volume, which was detected since gestational age of 5 months. According to the mother, fetal ultrasonography at that time was suggestive of renal malformation without exact details. Otherwise, he had been free of any significant illnesses including hepatitis B, tuberculosis, IgA vasculitis or systemic lupus erythematosus. His father had been diagnosed with “nephritis and kidney failure” at the age of 20 and had an allograft kidney transplantation for 10 years. Upon presentation, his vital signs and physical examinations, including eye examinations, were normal. The results of relevant investigations were depicted in Table 1. In summary, he had proteinuria, elevated levels of BUN and creatinine, hyperparathyroidism, acidosis and bilateral renal atrophy. Genetic study showed a heterozygous mutation in the *PAX*2 gene. Further studies on the family showed that the patient inherited the mutated gene from his father although no similar mutation was detected in paternal grandparents. The pedigree was shown in Fig. 1 and the gene mappings were shown in Fig. 2a and b.

**Table 1:** Relevant abnormalities in laboratory investigations

Laboratory Investigation	Results	Normal range
Urea nitrogen (mmol/L)	13.1~ 15.4	2.86–7.14
Creatinine (umol/L)	83.5~ 90.0	28.3 ± 6.2
Parathyroid Hormone (g/ml)	135.8	12.0–88.0
Blood CO_2_ level (mmol/L)	16.2	23–31
Renal ultrasonography (mm × mm)	Left: 53 × 21; Right: 49 × 25	Left:(65.5–73.5) × (32.7–36.9); Right:(60.7–68.3) × (32.3–35.7)
99mTc—DTPA	Whole: 45.42 ml/min: Right: 23.28 ml/min; left: 22.14 ml/min	
Gene study	heterozygous mutation point in *PAX2* c.418C > G (cytosine > guanine) chr10:102539262 p.R140G (arginine > glycine)

The child was managed medically by correcting the metabolic derangements secondary to chronic kidney diseases and by monitoring the progress. At last follow up at 1 year, all his initial metabolic changes normalized and his GFR did not deteriorate.

## Discussion and conclusions

CKD is a rapidly growing public health issue, affecting both children and adults globally. It brings physical suffering and inflicts financial burdens to those affected. While most of the adults with CKD are secondary to either hypertension or diabetes, the etiologies of CKD in children are much more heterogenous [5]. While CAKUT (congenital anomalies of kidney and urinary tract) are the main causes of CKD among children in developed countries, infection-related glomerulonephropathies seem to be the major culprits leading to CKD in the developing world [6]. It is significant to identify the primary disease of CKD, especially in children, which would play an important role to evaluate disease evolution and to guide treatment. Because of the good effect of kidney transplantation in patients with CKD caused by partial genetic factors, we should adequately understand the genotype and phenotype.

Our patient was at the age of 22 months when first presented, and he has already had metabolic acidosis, hyperparathyroidism and renal atrophy. He had an estimated GFR of 45 ml/min/1.73m^2^ and his symptoms had lasted for more than 3 months, which rendered him compatible to have a clinical diagnosis of CKD stage 3 [7]. Based on the fact that he had prenatal diagnosis of renal anomalies, reduced amniotic fluid and absence of other obvious etiology for CKD, in addition to having the same genetic mutation in the *PAX*2 gene as his father, who suffered from end stage renal diseases that required kidney allograft transplantation at early age, we concluded that his underlying cause of CKD was secondary to renal dysplasia associated with the *PAX*2 mutation (missense mutation, C to G transversion at position 418 in exon 4). The limitation of this approach is that the renal biopsy is not performed. Extend to the analysis to his grandparents, both of them have normal renal function and genotype. It can be concluded that the missense mutation c.418 C > G is a de novo mutation in the proband’s father, and the proband inherited the mutation from his father. Searching in a web site database for the human *PAX2* allelic variant (http://lsdb.hgu.mrc.ac.uk/pax2.txt), this mutation site has not been contained.

Development of mammalian metanephric kidney begins in early fetal life. The ureteric bud emerges from the Wolffian duct and extends outward towards the metanephros under the influence of trophic signals [8]. The *PAX2* gene is located close to the bands q24 and q25 on chromosome 10, and current data proves that it plays a pivotal role during kidney development. Mutations of *PAX2* gene in human and mice models have been associated with multiple renal anomalies, and the most commonly encountered malformation is renal hypoplasia [9]. *PAX2* is also expressed during the development of the eye, ear and nervous system. Although *PAX2* mutations have been reported mostly in patients with RCS, such mutations have also been identified in patients with congenital anomalies of these systems [10]. In a series of patients suffering from RCS with proven *PAX2* mutations, the renal, eye and high frequency hearing loss were found in 92%, 77% and 7% of patients, respectively [11]. Adam et al. have also reported a family with members suffering from RCS associated with heterozygous insertion mutation c.228_251dup and p.Ser77_Gly84dup, which had a wide spectrum of phenotypes [12].

The gene mutation site detected in this case has not been reported in the literature so far. Interestingly, our patient and his father both presented with chronic kidney disease without any optic involvement. Besides of renal function and complications of CKD, regular follow-up in ophthalmology and hearing test still will be done for him. We reviewed and summarized other reported cases with *PAX2* mutation leading to renal hypoplasia from 2000 to 2016, as depicted in Table 2, in order to observe reported phenotype and genotype of *PAX2* mutation, especially whether there is any optic involvement. As shown in Table 2, kinds of different *PAX2* mutations, including frame- shift, missense and nonsense mutations can be found in patients with RCS. Among these, Nishimoto et al. suggested that renal hypoplasia was part of the RCS that was caused by heterozygous mutations of the *PAX2* gene [13]. In their study, two novel nonsense mutations are reported. In addition, this is the first case of a *PAX2* mutation resulting in isolated renal hypoplasia without ocular involvement. Another patient with deletion in the region 10q23.2q24.3 on one of the chromosome 10, which led to complete deletion of one *PAX2* gene, has also had renal abnormalities without any ocular abnormality [14]. Furthermore, this is the first case of heterozygous *PAX2* gene deletion with renal abnormalities without optic coloboma in humans.

**Table 2:** Other reported cases with *PAX2* mutations in literature between the years 2000 to 2016

Patient	Age (years)	Renal hypoplasia	Renal insufficiency	Optic nerve colobama	VUR	*PAX2* mutation	Year	Reference
1	7	+	+	+	+	exon 2619 ins G	2000	[20]
2–1	8	+	+	–	+	exon 9 1566 C > A (novel)	2001	[13]
2–2	4	+	+	Right+;Left -	–	exon 7 1318 C > T (novel)	2001	[13]
3–1	6	+	+	+	unknown	832 del G	2001	[21]
3–2	37	+	+	+	unknown	832 del G	2001	[21]
3–3	12	+	–	+	unknown	619 ins G	2001	[21]
3–4	11	+	+−	+	unknown	619 ins G	2001	[21]
3–5	10	+	+−	+	unknown	619 ins G	2001	[21]
3–6	15	+	–	+	unknown	619 ins G	2001	[21]
3–7	19	+	+−	+	unknown	658–663 del	2001	[21]
3–8	17	+	–	+	unknown	619 ins G	2001	[21]
3–9	29	+	–	+	unknown	619 ins G	2001	[21]
4	9	+	+	+	unknown	exon 2602 del T (novel)	2001	[22]
5–1	34	+	+	+	–	exon 2755 G > C	2005	[23]
5–2	Mother of 5–1	+	+	+	–	exon 2755 G > C	2005	[23]
6–1	21, II-3	+	+	+	–	exon 2 (682–691) del CAGGGTGTGC	2005	[24]
6–2	3, III-3	+	+	–	+	exon 2 (682–691) del CAGGGTGTGC	2005	[24]
6–3	24, III-4	+	+	+	–	exon 2 (682–691) del CAGGGTGTGC	2005	[24]
6–4	3, IV-1	+	+	+	–	exon 2 (682–691) del CAGGGTGTGC	2005	[24]
6–5	0.5, V-2	+	+	+	–	exon 2 (682–691) del CAGGGTGTGC	2005	[24]
6–6	0.5, V-2	+	+	+	–	exon 2 (682–691) del CAGGGTGTGC	2005	[24]
7–1	21	+	+	+	–	exon 2619_620 ins G	2007	[25]
7–2	17	+	–	+	–	exon 2619_620 ins G	2007	[25]
7–3	11	+	–	+	–	exon 2619_620 ins G	2007	[25]
7–4	15	+	+	+	–	exon 2619_620 ins G	2007	[25]
7–5	2.5	+	–	+	–	exon 2619_620 ins G	2007	[25]
7–6	11	+	–	+	–	exon 3 p.R 104 ×	2007	[25]
8	5 month	+	+	–	–	del (10) (q23.2q24.3)	2007	[14]
9	19	+	+	+	+	exon 3 c.853 C > T (p.R104X)	2008	[26]
10	1 month	+	+	+	unknown	exon 2619 ins G	2010	[27]
11	<1	+	+	+	+	exon 2 del (10q24.2q24.32)	2012	[28]
12	8	+	+	+	–	exon 2 del (10q24.31)	2012	[29]
13	61	+	+	+	–	exon 3 c.228_251 dup	2013	[12]
14–1	unknown	+	+	+	unknown	exon 2 c.119–120 del GC	2015	[30]
14–2	unknown	+	+	+	unknown	exon 2 c.119–120 del GC	2015	[30]
14–3	unknown	+	+	+	unknown	exon 2 c.119–120 del GC	2015	[30]
14–4	unknown	+	+	+	unknown	exon 2 c.119–120 del GC	2015	[30]
14–5	unknown	+	+	+	unknown	exon 2 c.119–120 del GC	2015	[30]
14–6	unknown	+	+	+	unknown	exon 2 c.212G>C	2015	[30]
14–7	unknown	+	+	+	unknown	exon 2 c.212G>C	2015	[30]
14–8	unknown	+	+	+	unknown	exon 9 c.1023C>A	2015	[30]
14–9	unknown	+	+	+	unknown	exon 2 c.187G>A	2015	[30]
14–10	unknown	+	+	+	unknown	exon 2 c.57–58 ins GTGAACC	2015	[30]
14–11	unknown	+	+	+	unknown	exon 3 c.224-225insAC	2015	[30]
15–1	27	+	+	–	+	exon 2 c.76dupG (p.Val26Glyfs*28)	2016	[31]
15–2	2	+	+	–	–	exon 2 c.76dupG (p.Val26Glyfs*28)	2016	[31]
15–3	<1	+	+	–	–	exon 2 c.76dupG (p.Val26Glyfs*28)	2016	[31]

In our study, we reported a boy with heterozygous missense mutation of *PAX2*, that is C to G transversion at position 418 in exon 4, which changed arginine 140 to a glycine at the protein level. It is not clear how the change of molecular structure influences the function of gene. Talking about the structure of the gene, we have known that the *PAX2* paired domain consists of 2 globular protein sub-domains called N sub-domain and C sub-domain and a polypeptide chain between them. The *PAX2* N-subdomain (residues 16–74) contains 4 parts of structure as follows, an anti-parallel b-hairpin (residues 16–27) and 3 a-helices, a1 (residues 33–46), a2 (residues 49–57), and a3 (residues 58–74). Relatively, the C-subdomain also includes 3 a-helices, a4 (residues 88–105), a5 (residues 109–120) and a6 (residues 131–146) [15, 16]. AS reported, the C-subdomain is involved in protein-DNA interaction [15], and R140G in our study is located at a6, that may cause a change in the secondary structure of the a6-helix resulting in the loss of protein specificity. It can be inferred that a partially-functional or abnormally-functional protein product is made in vivo due to this kind of missense mutation, therefore the entire kidney development process is seriously affected.

Dressler et al. have done lots of researches to observe and prove the relationship between abnormal expression of *PAX2* and renal epithelial cells development [17, 18]. In addition, Ostrom et al. also proven the role that *PAX2* maintains cystic renal epithelia [19]. In light of the significant role of *PAX2* in nephrogenesis, it is possible that its failed expression in the mutant could contribute to renal hypoplasia with reduced number of glomeruli or complete renal agenesis causing decreased renal function even CKD.

Considering the function of filtration and incretion of kidney, the child was managed medically by correcting the metabolic derangements secondary to CKD (anemia, metabolic acidosis and hyperparathyroidism) and by monitoring the progress. Renal replacement therapy needs to be considered once reaching ESRD.

Therefore, current literature supports that *PAX2* mutations may have diverse clinical manifestations and renal hypoplasia is one distinct entity in the spectrum. Hence, genetic screening in patients with CAKUT is recommended. Although our patient is stable with medical therapy at this moment, he is being followed up closely to ensure timely intervention if his clinical condition changes.

## Abbreviations

CAKUT: Congenital anomalies of kidney and urinary tract
CKD: Chronic kidney disease
ESRD: End stage renal disease
GFR: Glomerular filtration rate
*PAX2*: Paired box 2
RCS: Renal coloboma syndrome
